# The Double Pedigree: A Method for Studying Culturally and Genetically Inherited Behavior in Tandem

**DOI:** 10.1371/journal.pone.0061254

**Published:** 2013-05-20

**Authors:** Etienne Danchin, Benoit Pujol, Richard H. Wagner

**Affiliations:** 1 CNRS, UPS, ENFA, EDB (Laboratoire Evolution et Diversité Biologique), UMR5174, Toulouse, France; 2 Université de Toulouse, EDB, UMR5174, Toulouse, France; 3 Konrad Lorenz Institute of Ethology, Department of Integrative Biology and Evolution, University of Veterinary Medicine Vienna, Vienna, Austria; Royal Holloway University of London, United Kingdom

## Abstract

Transgenerational sources of biological variation have been at the center of evolutionary studies ever since Darwin and Wallace identified natural selection. This is because evolution can only operate on traits whose variation is transmitted, i.e. traits that are heritable. The discovery of genetic inheritance has led to a semantic shift, resulting in the tendency to consider that only genes are inherited across generations. Today, however, concepts of heredity are being broadened again to integrate the accruing evidence of non-genetic inheritance, and many evolutionary biologists are calling for the inclusion of non-genetic inheritance into an inclusive evolutionary synthesis. Here, we focus on social heredity and its role in the inheritance of behavioral traits. We discuss quantitative genetics methods that might allow us to disentangle genetic and non-genetic transmission in natural populations with known pedigrees. We then propose an experimental design based on cross-fostering among animal cultures, environments and families that has the potential to partition inherited phenotypic variation into socially (i.e. culturally) and genetically inherited components. This approach builds towards a new conceptual framework based on the use of an extended version of the animal model of quantitative genetics to integrate genetic and cultural components of behavioral inheritance.

## Introduction

Ever since Darwin and Wallace identified natural selection, a central objective of evolutionary biology has been to evaluate the role of inheritance mechanisms on the evolution of phenotypic diversity. Darwin [1] underlined that evolution only affects traits whose variation is inherited and that phenotypic variation should be partitioned into inherited versus non-inherited components [2]. The discovery of genetic inheritance has tended to lead biologists to consider that genes alone are inherited across generations and play an exclusive role in evolutionary changes [3], [4]. As a consequence, phenotypic variation has been usually partitioned into genetic and non-genetic components in order to exclude non-genetic components from heritability estimates [5], [6]. However, evidence of non-genetic inheritance and heritability has been steadily accruing (reviews in [3], [4], [7]–[11]). Non-genetic inheritance encompasses several processes such as transgenerational epigenetic inheritance, [12]–[16], parental effects [17]–[21], ecological inheritance and niche construction [22]–[27], as well as cultural inheritance, which encompasses socially transmitted information [2], [8], [28]–[30] (Review in [3]).

Genetic and non-genetic inheritance are intricately interconnected and easily confounded [2], [3], [8]–[10], [31], [32]. It is common to find cases in which phenotypic variation that was initially thought to be genetically determined turned out to be non-genetically inherited. A well known example of this is the peloric form of toadflax (*Linaria vulgaris*) which was described more than 250 years ago by Linnaeus [33]. The change in symmetry of toadflax flowers (from bilateral to radial) was first attributed to genetic change but later shown not to involve mutations in the DNA sequence. In fact, this transition is due to a heritable change in gene expression through DNA methylation [33]. In another example, the natural tendency of cockroaches (*Periplaneta americana*) to flee light and move towards darkness is so strong and invariable in nature that it suggests genetic encoding. However, a study reported that this tendency can be reversed by social influences, suggesting that its determinism is plastic and that part of this tendency can be acquired socially, opening the way to social inheritance [34].

Thus, the long prevailing assumption that the inheritance of phenotypic variation rests exclusively on genetic variation is no longer tenable [3], [4] and predicting the evolution of phenotypic variation in response to natural selection, sexual selection and genetic drift should consider both genetic and non-genetic components of inheritance [2], [3], [7], [9], [10], [28], [35]. It is necessary to distinguish the effects of the various inheritance systems because the properties of their transmission modes differ in ways that should strongly affect evolutionary dynamics [3], [10]. For instance, whereas genetic, epigenetic, parental and ecological inheritance occur mainly vertically (i.e. between generation from parents to offspring), cultural inheritance in contrast, is frequently transmitted horizontally (i.e. among members of the same generation) as well as obliquely (i.e. between non-kin members of different generations).

Here, we focus on the vertical component of cultural inheritance, which can be used to calculate what we have termed the ‘inclusive heritability’ of a trait [2] similarly to Mameli [7] who coined ‘general heritability’. Inclusive heritability, which is in accordance with Darwin's view of inheritance and evolution, is a measure of transgenerational phenotypic variation that estimates the full potential for evolutionary change. Here, we first discuss current methods in quantitative genetics, and then propose an experimental design with the potential to estimate the impact of social (i.e. cultural) transmission on inheritance.

## Expanding Quantitative Genetics

### Methods to estimate the genetic and non-genetic components of heritability

Several methods exist that provide us with indirect tests to determine the inheritance of social information, for example the ‘Option-bias method’ [36], the ‘Randomization method’ [37], [38] and the ‘Network-based diffusion analysis’ [39]. However, these methods do not estimate the relative genetic and non-genetic components of phenotypic variation that are inherited vertically. Furthermore, the theoretical perspective adopted by Bonduriansky and Day [9] should permit the exploration of the impact of non-genetic inheritance on evolution by deriving the Price equation, which describes the relationship between fitness and phenotypic variation. Their approach allows the modeling of theoretical expectations on the evolution of non-genetically inherited phenotypic variation under natural selection. Finally, Otto *et al*. [40] developed path analysis methods for the same aim. The two latter approaches constitute powerful theoretical methods for modeling the evolution of inclusive heritability. There remains a scarcity of studies that propose a conceptual framework for the development of empirical approaches [3]. It is our aim to help fill this gap.

A method for decoupling genetic and non-genetic effects is the manipulation of progeny, such as cross-foster experiments, which entail exchanging offspring between families [41], [42]. For instance, by swapping eggs between nests of blue tits (*Cyanistes caeruleus*) and great tits (*Parus major*), two species with contrasting foraging niches, Slagsvold and Wiebe [42] recently showed that foraging habits, which are usually considered as genetically inherited, are in fact mainly transmitted socially from parents to offspring. We expect that such results will become common in the future as behavioral ecologists experimentally manipulate social information during development. Using cross-fostering methods represents a challenge that cannot be met for all biological groups for reasons of feasibility and ethics. However, some approaches have already proven successful in overcoming such limits. Combined with quantitative genetics approaches, cross-foster experiments should allow us to disentangle genetic and non-genetic components of inclusive heritability.

### Animal models

A promising opportunity to measure inclusive heritability and to evaluate the relative importance of genetic and non-genetic inheritance is the pedigree based “animal model” framework used in animal breeding and long-term studies of wild animal populations (reviews and methods in [43]–[45]). We now focus on the basic model but note that other derived models, such as the “variance component analysis” [46] or the “random regression model” [47]–[49], also offer promising options. The advantage of the animal model is that it partitions phenotypic variation into genetic and non-genetic components [43]–[45], [50], [51]. It would certainly not be trivial to account for social effects that occur prior to birth in any study because they are not easily manipulated. However, it would be similar in terms of statistical analysis to quantifying permanent environmental and/or maternal effects in the animal model framework. For example, studies of embryo transfer in mammals (horses) are known that would allow us to test for such effects [52].

The animal model approach is a pedigree-based mixed linear model of variance that is one of the most advanced methods in quantitative genetics for studying populations with known pedigrees (reviews and methods in [43]–[45]). The number of researchers using the animal model is increasing because the approach considerably improves our capacity to estimate genetic heritability by accounting for extended relatives over multigenerational pedigrees in monitored wild populations. In its simplest form, the model can be written as
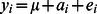
where *y* is the measure of a continuous trait characterizing an individual *i*, *μ* is the average phenotypic value of the population, *a*_i_ is the additive genetic component of an individual *i* to transgenerational phenotypic variation and *e* is the residual error. The total phenotypic variance in *y* is the phenotypic variance *σ* ^2^_P_. Random effects *a*_i_ are defined as having a variance equal to *σ* ^2^_A_, i.e. the additive genetic variance of the trait, with *e*_i_ defined as having a variance equal to *σ* ^2^_R_, i.e., the residual variance. Fixed effects can be fitted in the model to estimate the impact of a particular type of environmental stress on phenotypic variation. Other random effects can be included in the model to estimate factors such as maternal and (permanent) environmental effects on phenotypic variation [43]–[45], [50].

This approach is the best way to incorporate all non-genetic components of variation, including culture, in empirical estimates of heritability. The first and simplest approach we propose consists of defining a random variable in the model that represents the cultural environment of an individual. This variable can take different forms (from binary to continuous) as long as its variation is not confounded by genetic or environmental variation. Cross-fostering experiments should allow us to statistically test the significance of the effect of such a random variable and to estimate its effect size. As a result, the impact of cultural changes on phenotypic variation would be known. Adding the effect of an individual's birth place in an animal model makes it possible to measure the effect on phenotypic variance of the common environment shared by a family, part of which may be cultural. Although such an effect can be used to measure common brood and long-term environmental effects, it is not suitable for quantifying the extent to which the inheritance of a behavioral trait is socially mediated, i.e., to estimate cultural heritability, because cultural variation occurs among populations and not among families. In humans for instance, statistical settings based on twins are used similarly to cross-fostering experiments to estimate the heritability of traits such as cognitive capacity [53], [54]. The within-pair comparison of identical twins raised in different cultural environments provides a tool for estimating the respective roles of shared genes (plus inherited epigenetic differences) versus a shared cultural environment. Similarly, we propose to use the animal model as a framework for performing empirical studies of the genetic and non-genetic components of inclusive heritability.

### Genetic and cultural pedigrees

The key to understanding why the animal model framework is suitable to estimate and partition inclusive heritability lies in the use of the pedigree. The pedigree of the population recapitulates the genealogy among all individuals in the population (note: details on building and implementing pedigrees can be found in [45], [50]). The pedigree is used to estimate Θ_ij_, the coefficient of coancestry between individuals *i* and *j*. Θ_ij_ is the probability that one allele chosen randomly in individual *i* is identical by descent to an allele chosen randomly in individual *j*. On the basis of Θ_ij_, we can then calculate the additive genetic covariance between *i* and *j*, which is calculated by doubling Θ_ij_: *A_ij_* = 2Θ_ij_. Because *Aij* can be calculated for all pairs of individuals in the pedigree, it is possible to build the additive genetic relationship matrix A for the entire population. Solving an animal model consists of obtaining the additive genetic variance *σ* ^2^_A_ from the covariation between the matrix **A** and phenotypic variation [43], [55]. Narrow sense heritability *h* ^2^ is the part of a trait's phenotypic variation that is determined by additive genetic variation: *h* ^2^ =  *σ* ^2^_A_/*σ* ^2^_P_. The additive variance component *σ* ^2^_A_ is therefore the key parameter for the empirical estimation of heritability.

As stated in the previous section, it is possible to include a cultural environment as a random variable corresponding to the culture in which natal and cross-fostered individuals were raised. A second and more promising approach may consist of building a matrix **C** that summarizes the cultural relationships between all pairs of individuals in the population. Similarly to how the matrix **A** is built on the basis of the genealogical pedigree, we propose that the **C** matrix could be built on the basis of a ‘cultural pedigree’ which we describe as ‘lineages’ of segregating cultures that sum up to the culture in which an individual is raised. Associated transgenerational effects would then be tested by evaluating the covariation between phenotypic variation and cultural resemblance. As a non-genetic parallel to *a_ij_*; the term *c_ij_* in the cultural matrix **C** would thus represent the coefficient of ‘cultural coancestry’ between individuals *i* and *j*.

Depending on the study system, the cultural pedigree could take the form of a social network, comprising for instance a successive suite of teachers and pupils, as long as it reflects the transgenerational transmission between any pair of individuals resulting from their social interactions, independently from kinship. A simplification of the cultural pedigree could be to build **C** directly on the basis of the amount of shared information between pairs of individuals. For example, a matrix of social encounters was used in a recent study of the transmission of behavior in a non-manipulated population of wild dolphins in Western Australia [56]. The researchers used observations of pairwise interactions between individuals to build an equivalent of our **C** matrix of social relationship and used it to estimate the role of social relationships on behavioral inheritance. They compared that model to an alternative one that only accounted for the role of quantitative genetic variation estimated from genetic relatedness using 12 microsatellite markers (which might be insufficient to measure genetic segregation [56]). They found that female calving success depended on both genetic inheritance and social bonds. This is an innovative approach but it remains necessary to go further by building a model that includes both **C** and **A** to disentangle the social and the genetic component of inheritance.

It is important to note that although the statistical tool-box of pedigree-based animal models is quite accessible its use requires researchers to have a background in quantitative genetics. When applied to non-experimental data, an extended animal model incorporating both genetic and cultural pedigrees would probably lack sufficient analytical power to separate the effects of the two inheritance systems [43], [44]. As we discuss in the next section, this lack of statistical power stems from the fact that in unmanipulated populations, the Matrices **A** and **C** are practically identical.

### Resolving the issue of overlapping pedigrees

A major difficulty in partitioning heritability stems from the risk that different inheritance systems may be confounded within vertical transmission. Because social heredity mainly depends on parent-offspring relationships, in non-experimental data we expect vertical links in the genetic and cultural pedigrees to be almost identical in the absence of extrapair paternity, intraspecific brood parasitism and adoption. This overlap in cultural and genetic informational pathways leads the statistical models to capture the vertical component of variation due to both genetic and cultural inheritance. Despite this, we typically interpret the statistical estimate of the vertical transmission exclusively in terms of genes [57]. However, it is necessary to keep in mind that this is only one possible interpretation of a statistical term.

Dispersal can lead to the dissemination of culture as long as dispersers move sufficient distances to change their cultural groups. Exchanges of individuals, or genes, occur between groups but it is uncommon in most animal species for females to leave their natal population (and potentially cultural group) when seeking extrapair, or to lay parasitic eggs, and return. Another problem stems from the potential inaccuracy of pedigrees because most often observational data is blind to extrapair paternity, adoption or brood parasitism. Several studies have dealt with this issue [58], and a software program (the module RPEDERRBIRD in the software PEDANTICS [59]) has been designed to anticipate the possible biases produced by extrapair paternity (or more generally, pedigree errors) on estimates of genetic heritability. Nevertheless, even if extra pair paternity dispersal and adoptions were detected by using DNA fingerprinting, it remains unclear whether the genealogical structure of natural populations can be satisfactorily uncoupled from their social or cultural structure.

For instance, genetic and cultural pedigrees can be confounded in the process of offspring learning their first language, which is mainly transmitted by parents. Estimates of the genetic inheritance of language calculated from data on sedentary individuals would misleadingly incorporate the part of language variation that is inherited vertically, but non-genetically, by social learning. The same reasoning holds for song learning in birds, whales and dolphins [60]–[64]. Exchanges between genetic families and social environments via cross-fostering experiments are necessary to test whether genes are expressed differently under different cultural environments. Including ‘country’ as a common language environment factor in such analysis would not solve this issue because of colinearity problems between the country and cultural factors.

Despite the fact that heritability estimates of behavioral traits may include substantial overlapping information from genetic and cultural pedigrees [57], several studies have concluded that such estimates were due to genetic transmission only without considering the potential contribution of socially acquired variation. For instance, Haesler and Seehausen [65] performed an experiment to test for the transmission of mating preferences in two sympatric sister species of the cichlid fishes *Pundamilia pundamilia* and *P. nyererei*. They concluded that female mating preferences were heritable and discussed the supposed genetic system involved in the inheritance of this behavior. However, given that parents provide care to their offspring, the experiment did not exclude the possibility of cultural transmission through sexual imprinting early in life. A second study that cross-fostered fry between the same sister species resulted in the reversal of each species' preference for its own kind [66]. This finding suggests that female mating preferences derive substantially from early social imprinting and that the divergence of these two species stems partially from cultural divergence [66]. A third study suggested that genetic and cultural inheritance may interact in isolating the two sympatric sister species of cichlid fish in Lake Victoria [67], [68]. It is important to note that one advantage of the animal model framework is that “gene-by-culture interactions” can be tested by following the methodology used for testing gene-by-environment interactions.

Similarly, a study of a wild population of western bluebirds (*Sialia mexicana* [69]) provided the first evidence of ‘heritable’ variation in helping at the nest. Narrow sense heritability was surprisingly high (0.76) for such a complex behavioral pattern. Although analyses controlled for ecological inheritance, and showed that it influenced helping patterns, the possibility that offspring were socially imprinted on their parents' behavior was not excluded. As acknowledged by the authors [69], the genetic component might thus have captured part of cultural inheritance. Those authors thus concluded that “To clearly distinguish genetic from cultural inheritance, future studies would need to carry out multigenerational cross-fostering experiments”. Cross-fostering experiments are particularly powerful tools to uncouple types of pedigrees because very young individuals with similar genotypes can be raised in contrasting cultural environments from those of their genetic parents.

More generally, several reviews provide evidence of great potential for social information to affect behavioral inheritance across a wide range of animal taxa (eg [28], [70]–[77]). This implies that the cultural component of inclusive heritability should always be taken into account in the measurement of the inclusive heritability of behavior. We now offer and discuss an experimental design aimed at estimating the cultural component of the inclusive heritability of a behavioral trait.

### The double pedigree

The double pedigree experiment is the sum of a cross-fostering experiment coupled with an animal model analysis. In this approach, one random effect takes into account the additive genetic relationship extracted from the genealogical pedigree of the population and a second random effect takes into account the cultural relationship between individuals. Because such an approach is time consuming, we first suggest precautions that may be adopted before starting the protocol.

### Choice of behavioral trait

A way to increase the odds of finding a meaningful cultural component of inheritance is to identify a behavioral trait that fulfills criteria that demonstrate a trait is (at least partially) culturally inherited [2] and that shows among-group variation that persists over generations. Each group of individuals showing the same behavior will then constitute a particular culture. The goal of the experiment is to quantify the different components that explain among-group behavioral variation.

Accounting for epigenetic transmissibility and environmental inductions can also allow the estimation of the epigenetic component of inclusive heritability [32], part of which may result from cultural inheritance. This approach requires that researchers know the parents, uncles and siblings (sibs) of each focal individual. Thus, it uses parts of pedigrees. As with the animal model, the strength of the double pedigree approach is that it uses all possible degrees of kinship between members of the study population, allowing us to estimate genetic or non genetic phenotypic covariance among all pairs of individuals and therefore more accurately evaluate the genetic component of inclusive inheritance.

### The experimental design

**Figure 1:**
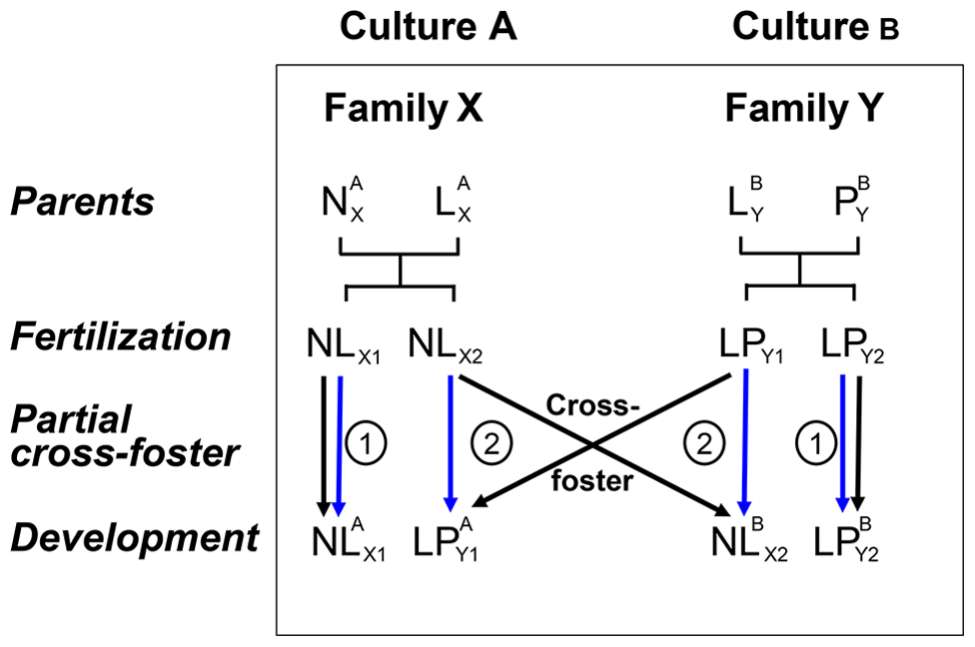
A cross-fostering experiment uncouples the cultural from the genetic pedigree to apportion the genetic and cultural components of behavioral traits. Note that L^A^ and L^B^ are siblings that were cross-fostered between cultures A and B. *Superscript*: cultures; *subscript* family of origin, plus identity of the offspring. *Black arrows*: genetic genealogy; *Blue arrows*: cultural genealogy. Cross-fostering should be performed as early in life as possible to avoid any social influence. According to this protocol, half of the offspring remain in their nests of origin (NL_X1_ and LP_Y2_). Their genetic (black) and cultural (blue) genealogies are thus confounded (arrows labeled 1). In contrast, for the other half of the offspring (NL_X2_ and LP_Y1_) the cross-fostering uncouples the genetic (black) from the cultural (blue) genealogy (arrows labeled 2). This allows us to differentiate the respective roles of genetic versus cultural inheritance in resemblance. The comparison of cross-fostered versus non-cross-fostered siblings allows the partitioning of variance between genetic and cultural effects. It is thus crucial to perform partial cross-fostering in which only some of the siblings are cross-fostered.

The double pedigree experimental design uses partial cross-fostering manipulations between identified behavioral groups to uncouple the genetic and cultural sources of phenotypic variation of behavioral traits (Figure 1). This is achieved because siblings are raised in contrasting cultural environments independently from that of their genetic parents. This should allow us to quantify the cultural component of inclusive heritability.

**Figure 2:**
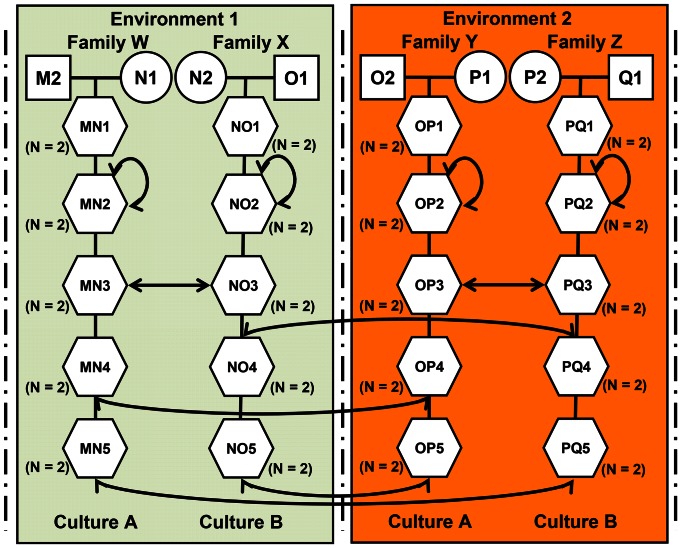
The ideal double pedigree protocol to study the interactions between genotype, environment and culture. This can be simplified in the lab by providing only one type of environment. Each column represents one family and hence one set of parental genes reorganized in different individual genotypes. The colored boxes represent different environments. O_1_ and O_2_ are siblings. This illustrates the possibility of linking families across environments. Squares and circles are male and female parents and hexagons are offspring. In this design we consider that there are two identified cultures (e.g. song dialects or languages). Sample sizes are set at two which is the minimum necessary to account for residual variance. Two-way arrows indicate partial cross-fostering among environments, families and cultures. The arrows starting and ending on the same family corresponds to controls for the effect of the manipulation where eggs or young are handled, moved over a comparable period of time, then put back in their original nest or habitat. In this cross-foster design all combinations of environment, genotype and culture can be created and replicated. Ideally, some of the cross-fosterings are performed within the same culture/environment/family to test for the manipulation effect. The cross-fosters are then used to build the matrix of cultural distances among individuals, which describes the cultural pedigree (see text). An advantage of the animal model is that it is robust enough to cope with unbalanced designs resulting from the unavoidable death of some individuals.

Partial cross-fostering uncouples sources of genetic and cultural variance by fostering half of newly born clutches, litters or broods between genetic and cultural environments (i.e. a place where individuals can learn from others, which can be their birth place or a location to which they have been experimentally transferred). Because social influences can occur very early in development [78] it is wise to transfer offspring at the youngest possible age, for instance, at the egg stage in oviparous species [42]. It is crucial that some individuals within a lineage are kept unmanipulated to serve as controls in the pedigree-based analysis whereas others from the same lineage are cross-fostered between different cultures to dissociate cultural and genetic effects (Figure 2). It is also necessary to cross-foster some individuals within the same culture to control for the manipulation effect (Figure 2). Details of the design need to be adapted to the focal behavioral trait and to the biological characteristics of the species.

Various techniques may be used to avoid confounding common environment or parental effects with the effect of culture. For instance, by using a species with multiple offspring in a single reproductive event, it may be possible to foster several sibs in different families of the same and different cultural groups. This could be performed over several generations so that the variables capturing the variability of cultures, common brood (by foster family) and parental effects (maternal effects) would be independent given that genetically related individuals are spread across those factors. Our purpose is to separate shared culture from genetic resemblance. As a result of the design proposed in Figure 2, the expanded animal model could be written as:
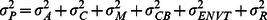
where *σ^2^*_P_ is the total phenotypic variance, *σ^2^*_A_ is the additive genetic variance, *σ^2^*_C_ is the additive cultural variance, *σ^2^*_M_ is the maternal effect variance, *σ^2^*_CB_ is the common brood effect variance, *σ^2^*_ENVT_ is the environmental variance and *σ^2^*_R_ is the residual variance. In this model, partitioning inclusive heritability between the cultural and genetic components is possible: *σ^2^*_A_/*σ^2^*_P_ would estimate the narrow sense genetic heritability and *σ^2^*_C_/*σ^2^*_P_ the cultural component of inclusive heritability. Such experiments could be performed in animals with vocal dialects, particularly in species that are easy to manipulate, such as hole-nesting birds. This design also avoids the developmental problems resulting from social deprivation because by being fostered in another cultural environment, rather than being deprived of social stimulation, cross-fostered young should develop normal social learning capacities.

### Analyses

The resulting data should be analyzed in a model incorporating both genetic and social pedigrees simultaneously. In this multivariate model, sources of phenotypic variation would thus be partitioned between their genetic and cultural components and their interaction. The genetic component is estimated by the covariance between trait variation and genetic relatedness, while the cultural component would be estimated by the covariation between trait variation and cultural relatedness as defined above. The cross-fostering experiment allows partitioning of phenotypic variation between these two sources of trait variation by uncoupling the (usually largely) overlapping pedigrees.

In this model the information derived from the genetic pedigree takes the form of the matrix of genetic relatedness **A**. The information derived from the cultural pedigree (included in the model as a random variable) may take various forms: 1) the identity of the cultures between individuals that were cross-fostered, which is an individual characteristic, 2) the pairwise sum of social contacts in the form of a matrix **C**, and 3) the vertical component of socially transmitted information built on the basis of the transgenerational segregation of different cultures that would account for the complex nature of culture as defined by the analyst (i.e., it could take the form of the previous pairwise matrix of social encounters multiplied by a vector of social inertia, the weighting of which could be modified according to the types of encounters (e.g. between grand parent/grand children, competitors, helpers, etc.)).

The value of these models is not restricted to detecting cultural inheritance. It is also in the opportunity to compare the phenotypic variation explained by genetic relatedness, cultural transmission and the inclusive genetic and cultural determinism. This can be done by comparing the goodness of fit of those models with parameters such as the Akaike Information Criterion (AIC) for maximum likelihood tests or the Deviance Information Criterion (DIC) for a Bayesian Markov Chain Monte Carlo approach [45]. It is important to note that the double pedigree approach also allows for testing whether the interaction between cultural and genetic relatedness affects the variance of the behavioral trait of interest.

The approach developed by Slate *et al*. [46], [79], [80] to map Quantitative Trait Loci (QTLs) in a wild population of ungulates, can be used to incorporate social information by replacing the matrix that encodes genetic mapping information with a matrix that encodes cultural relationship information. Slate's model to analyze variance components incorporates a random effect describing a polygenic effect in a statistical model derived from the animal model. We suggest adapting this method by integrating a matrix **C** that recapitulates the information gained from experimentally manipulated cultural pedigrees in place of Slate *et al*.'s genomic data [46], [79], [80]. For instance, recent analytical developments, encompassing genetic correlations as well as interactions between genes and the environment in the wild [81], provide an opportunity to study local adaptation. The same logic can be applied to study how genetic architecture and cultural inheritance interact in microevolutionary processes. One of the most exciting recent developments in quantitative genetics is the study of how genes interact with other genes, environments and age because such interactions alter the evolutionary trajectory traced by lineages (see discussion in [82]). The next step is to integrate the role of cultural components of variance into the equation.

It is important to note that in a partial cross-foster design, the comparison of the variance among true sibs raised in different cultural environments with that of foster-sibs in the same cultural environment can allow us to disentangle the cultural component from the genetic component of inclusive heritability. Thus, the classical two dimensional cross-fostering design (relatedness × environment) will become more complex with the addition of a third dimension, that of cultural relationships (Figure 2). Such practical challenges may be overcome by using a model species with multiple offspring per reproductive event that can be cross-fostered in different environments and cultures early in life (Figure 2). The dissection of common brood effects due to genetic, environmental and cultural factors will allow us to compare the stability across time of each of these factors. It is possible to build the **G** matrix, which defines how microevolutionary parameters (i.e., additive-genetic variance-covariance matrix) change over time and across environments [83]. We propose to build a similar type of matrix corresponding to parameters of cultural inheritance: the ‘**CVC** matrix’ (i.e., cultural variance-covariance matrix). We will then be able to compare the role and stability of these genetic and cultural links across time and environments. For instance, language inheritance and mate choice copying experiments illustrate how such cross-foster experiments constitute a powerful tool that could be used to estimate the inclusive heritability of these traits and disentangle their cultural and genetic determinism (figures 1 and 2). We encourage the use of these methods to unravel the complexity of inclusive heritability.

## Conclusions

Evolutionary biologists seem to periodically rediscover that, by concentrating on the genetic component of inheritance, we are missing the phenotypic, environmentally induced component of inclusive heritability. This fundamental statement was first formulated four decades ago by Anthony D. Bradshaw [84]. We think that the time is ripe to incorporate every form of inheritance into our evolutionary reasoning [3]. A first step is to quantify the relative weight of all components of inclusive heritability in the shaping of phenotypic variation. We have focused here on the cultural component. We propose a conceptual framework that uses animal model quantitative genetics methods to explicitly assess the role of cultural transmission in the evolution of behaviors, especially since many of those are already suspected to be at least partly inherited culturally. More generally, similar methods can be used to study the inheritance of all behavioral traits or behavioral syndromes and personalities.
